# Towards the PCR-based identification of Palaearctic *Culicoides* biting midges (Diptera: Ceratopogonidae): results from an international ring trial targeting four species of the subgenus *Avaritia*

**DOI:** 10.1186/1756-3305-7-223

**Published:** 2014-05-14

**Authors:** Claire Garros, Thomas Balenghien, Simon Carpenter, Jean-Claude Delécolle, Rudy Meiswinkel, Aurélie Pédarrieu, Ignace Rakotoarivony, Laetitia Gardès, Nick Golding, James Barber, Miguel Miranda, David Borràs Borràs, Maria Goffredo, Federica Monaco, Nonito Pagès, Soufien Sghaier, Salah Hammami, Jorge H Calvo, Javier Lucientes, Dirk Geysen, Gill De Deken, Victor Sarto i Monteys, Jan Schwenkenbecher, Helge Kampen, Bernd Hoffmann, Kathrin Lehmann, Doreen Werner, Thierry Baldet, Renaud Lancelot, Catherine Cêtre-Sossah

**Affiliations:** 1 Cirad, UMR15 CMAEE, 34398 Montpellier, France; 2 INRA, UMR1309 CMAEE, 34398 Montpellier, France; 3 The Pirbright Institute, Pirbright, UK; 4 Faculté de Médecine de Strasbourg, IPPTS, Strasbourg, France; 5 CIDC, Central Institute for Animal diseases, Lelystad, Netherlands; 6 Laboratory of Zoology, UIB, University of Balearic Islands, Palma de Mallorca, Spain; 7 IZS, Istituto Zooprofilatico Sperimentale dell’Abruzzo e del Molise “G Caporale”, Teramo, Italy; 8 Centre de Recerca en Sanitat Animal (CReSA), UAB-IRTA, Campus de la Universitat Autònoma de Barcelona, Bellaterra (Cerdanyola del Vallès), Spain; 9 IRVT, Institut de la Recherche Vétérinaire de Tunisie, Université Tunis El Manar, Tunis, Tunisia; 10 CITA-ARAID, Centro de Investigacion y Tecnologia Agroalimentaria de Aragon, Zaragoza, Spain; 11 UZ, Facultad de Veterinaria, Universidad de Zaragoza, Zaragoza, Spain; 12 Institute of Tropical Medicine, Antwerp, Belgium; 13 Institut de Ciència i Tecnologia Ambientals (ICTA), Bellaterra, Spain; 14 IBES, Institute of Biological and Environmental Sciences, University of Aberdeen, Aberdeen, Scotland; 15 FLI, Friedrich-Loeffler Institut, Greifswald-Insel Riems, Germany; 16 Humboldt-Universität zu Berlin, Cytogenetics, Chausseestr 117, Berlin, Germany; 17 Leibniz Centre for Agricultural Landscape Research (ZALF), Müncheberg, Germany

**Keywords:** *Culicoides*, Subgenus *Avaritia*, Obsoletus group, Molecular identification

## Abstract

**Background:**

Biting midges of the genus *Culicoides* (Diptera: Ceratopogonidae) are biological vectors of internationally important arboviruses. To understand the role of *Culicoides* in the transmission of these viruses, it is essential to correctly identify the species involved. Within the western Palaearctic region, the main suspected vector species, *C. obsoletus, C. scoticus, C. dewulfi* and *C. chiopterus*, have similar wing patterns, which makes it difficult to separate and identify them correctly.

**Methods:**

In this study, designed as an inter-laboratory ring trial with twelve partners from Europe and North Africa, we assess four PCR-based assays which are used routinely to differentiate the four species of *Culicoides* listed above. The assays based on mitochondrial or ribosomal DNA or microarray hybridisation were tested using aliquots of *Culicoides* DNA (extracted using commercial kits), crude lysates of ground specimens and whole *Culicoides* (265 individuals), and non-*Culicoides* Ceratopogonidae (13 individuals) collected from across Europe.

**Results:**

A total of 800 molecular assays were implemented. The in-house assays functioned effectively, although specificity and sensitivity varied according to the molecular marker and DNA extraction method used. The Obsoletus group specificity was overall high (95-99%) while the sensitivity varied greatly (59.6-100%). DNA extraction methods impacted the sensitivity of the assays as well as the type of sample used as template for the DNA extraction.

**Conclusions:**

The results are discussed in terms of current use of species diagnostic assays and the future development of molecular tools for the rapid differentiation of cryptic *Culicoides* species.

## Background

International multi-centre ring trials (IMRT) are used in many fields to assess the sensitivity, specificity and performance of diagnostic tools particularly in reference laboratories. In animal health surveillance, IMRTs have been organized to assess the detection of a wide range of pathogens including *Trypanosoma*, *Leishmania*, *Trichinella*, bluetongue virus and porcine circovirus [1-6]. To date no such IMRTs have been conducted to assess the various molecular identification assays now being used increasingly around the world to distinguish between an ever-growing number of morphologically cryptic arthropod species that are often differentially involved in pathogen transmission. Recently, a comparative analysis of four widely used molecular identification assays [7] highlighted discrepancies and demonstrated that the two common molecular assays utilized to differentiate between the M and S forms of *Anopheles gambiae* (Meigen) are not fully interchangeable. Similarly, successful vector control relies on accurate information concerning the insect populations being targeted in order to choose the most appropriate intervention method to be adopted and to monitor its impact.

*Culicoides* biting midges are the primary biological vectors of arboviruses such as bluetongue virus (BTV), African horse sickness virus (AHSV), and epizootic haemorrhagic disease virus (EHDV) [8]. Since 1998, bluetongue has emerged in Europe as an economically important disease affecting livestock, with the number and extent of outbreaks increasing substantially, both within the Mediterranean basin and in northern Europe [9]. More recently, a novel *Culicoides*-borne virus, provisionally named Schmallenberg virus (SBV), has also emerged in Europe and spread rapidly over a large area lying between Scandinavia and southern Europe [10-12].

*Culicoides* species of the subgenus *Avaritia* are thought to be the primary vectors of BTV and SBV north of the Mediterranean region, based on abundance and host preference [13-16], vector competence studies [17-19], and isolation or detection of virus in field-collected midges [15,20-26].

In the western Palaearctic region, the subgenus *Avaritia* is subdivided further into species groups or species complexes that have never been tested for monophyly. Moreover, the use of the subgenus *Avaritia* as a taxonomic entity presents with a confused history, with some workers preferring to use instead the more informal *C. obsoletus* group or complex instead [27]. There is clearly a lack of consensus in the grouping names, and phylogenetic data are required to solve the phylogenetic relationships within the subgenus *Avaritia* and its groupings. Today, the subgenus *Avaritia* within the Western Palearctic consists of at least five species and that these include *C. imicola* Kieffer, and the Obsoletus group, which according to author, varies from 2 to 4 species: *C. obsoletus* (Meigen), *C. scoticus* Downes and Kettle, *C. dewulfi* Goetghebuer and *C. chiopterus* (Meigen)*,* all widely distributed and sympatric over western and northern Europe [15,16,27]. The exclusion of *C. dewulfi* as an Obsoletus group species has been recently suggested based on wing shape [28] and phylogenetic studies [29]. We refer in this study to the Obsoletus group as an unformal grouping with no phylogenetic basis, which includes *C. obsoletus*, *C. scoticus*, *C. chiopterus* and *C. dewulfi*.

While male members of the subgenus *Avaritia* in western Europe can be identified reliably based upon marked differences in their genitalia, the routine identification of the females is less straightforward [30-32]. In a large majority of cases, *C. chiopterus* can be distinguished on its pale wing markings and small size, while the shape of the abdominal tergites in *C. dewulfi* are characteristic [30]. The accurate separation of *C. obsoletus* and *C. scoticus*, based on morphology alone, present the greatest challenge. These species are commonly grouped as the *C. obsoletus* complex [27,30,33]. While traditional and geometric morphometrics can be used to differentiate females of these two species of the *C. obsoletus* complex [28,30,34], several studies have highlighted phenotypic variation in characters used and these techniques require slide mounting of specimens in most cases which is very time-consuming and laborious [30,34,35].

As a consequence, in the last decade, a number of PCR-based identification assays have been developed to overcome the limitations that surround the accurate morphological identification of species within the subgenus *Avaritia* in particular within those that comprise the Obsoletus complex and the related species. Six PCR-based molecular assays have been described, using either ribosomal or mitochondrial DNA as the target [31,36-40], but at the time of the current study, only four of these assays had been published and were therefore assessed (Table 1). These assays are currently used independently by several research institutes to conduct studies on host-vector contact, larval ecology, vector competence, insecticide susceptibility, seasonal dynamics and spatial distribution; the conclusions drawn from such molecular-based investigations therefore have a great modernizing impact on our understanding of *Culicoides*-borne virus transmission. An IMRT was therefore organised in the framework of the European MedReoNet project (Surveillance network of Reoviruses in the Mediterranean basin and Europe) to assess the accuracy and sensitivity of molecular identification assays for differentiating amongst four of the species that comprise the subgenus *Avaritia* within the western Palaearctic region.

**Table 1:** Molecular identification assays published for the Obsoletus group in the Palaearctic region and used during the ring trials

**Reference**	**Identified species**	**Molecular marker used**^**1**^	**Type of assay**
[39]	*C. obsoletus, C. scoticus, C. chiopterus, C. dewulfi*	COI	Species-specific multiplex PCR
[37]	*C. obsoletus, C. scoticus, C. chiopterus, C. dewulfi, C. montanus*	ITS1	Species-specific multiplex PCR
[37,44]	*C. obsoletus, C. scoticus, C. chiopterus, C. dewulfi*	ITS2/ITS1	Species-specific multiplex PCR
[36]	*C. obsoletus, C. scoticus, C. chiopterus, C. dewulfi*	ITS1	Microarray

^1^COI : Cytochrome Oxidase 1, ITS1 : Internal Transcribed Spacer 1, ITS2 : Internal Transcribed Spacer 2.

## Methods

### Diversity and origin of culicoides used

To conduct the trials, 278 specimens (265 *Culicoides* and 13 non-*Culicoides*) were used from 5 European countries (Table 2). These specimens were collected during surveillance activities and stored in 70% ethanol prior to use. Two international expert taxonomists (JCD and RM) morphologically identified 48 of the 224 individuals belonging to the Obsoletus group (24 males and 24 females) using a double-blind procedure to assess the accuracy between the identifications made respectively by two taxonomists experienced in the morphology of Western European *Culicoides.* Thus, 24 individual midges identified by JCD were sent as blind samples to RM for him to identify and who, in turn sent 24 other individuals to JCD. *Culicoides chiopterus* and *C. dewulfi* were morphologically distinguished based on the wing pattern and spermatheca size for the females, while *C. obsoletus* and *C. scoticus* were separated by the experts based on the shape of the chitinuous plates although this method was found to not be 100% reliable [35]. All other specimens used in the performance trial were morphologically identified by one of the experts (JCD) and included 26 *C. imicola* Kieffer; 13 *C. newsteadi* Austen; 1 *C. pulicaris* (Linnaeus); 1 *C. nubeculosus* (Meigen) and 13 non-*Culicoides* Ceratopogonidae (13 *Forcipomyia bipunctata* (Linnaeus)).

**Table 2:** Number and origin of specimens belonging to the Obsoletus group included in each of the three ring trials (RT)

**Species**	**No. and origin of specimens**^**1**^	**RT1**	**RT2a**	**RT2b**	**RT3**
		**Ground midge**	**DNA sample**	**DNA sample**	**Whole midge**
*Culicoides chiopterus*	12 BE, 21 FR, 6 NL, 17 GB	4 ♀ + 3 ♂	1 ♂	2 ♀ + 2 ♂	44 ♀
*Culicoides dewulfi*	14 BE, 22 FR, 6 NL, 1 CH 12 GB	3 ♀ + 4 ♂	1 ♀	2 ♀ + 2 ♂	43 ♀
*Culicoides obsoletus*	12 BE, 19 FR, 6 NL, 1 CH, 19 GB	4 ♀ + 3 ♂	1 ♂	2 ♀ + 2 ♂	45 ♀
*Culicoides scoticus*	15 BE, 28 CO, 6 FR, 6 NL, 1 GB	3 ♀ + 4 ♂	1 ♀	2 ♀ + 2 ♂	44 ♀
*Culicoides imicola*	26 FR	2 ♀		2 ♀	22 ♀
*Culicoides newsteadi*	13 FR	1 ♀		1 ♀	11 ♀
*Culicoides pulicaris*	1 FR	1 ♀			
*Culicoides nubeculosus*	1 FR	1 ♀			
*Forcipomyia bipunctata*	13 FR	1 ♀		1 ♀	11 ♀

^1^BE: Belgium, FR: French mainland, CO: Corsica, NL: the Netherlands, CH: Switzerland, GB: United-Kingdom.

### Ring trials

The sensitivity and specificity of the molecular assays were assessed using three separate ring trials (RT1; RT2a; RT2b; RT3). Participating laboratories used their in-house protocols and filled out a form detailing the procedure used, recording DNA extraction method used; molecular assay performed and specific molecular marker targeted (Table 3). The three trials were differentiated based on the sample type sent by the coordinator laboratory (ground midge for RT1; DNA sample for RT2 and whole midge for RT3). For the first ring trial (RT1, ground midge trial), 28 specimens were individually ground using a pellet pestle in 200 μL of 1x phosphate-buffered saline (PBS) (Sigma-Aldrich, St Louis, MO, USA). From each sample, one subsample of 11 μL of lysate was sent to each participating laboratories with randomised labelling, the day after the grinding. For the second ring trial (RT2a and RT2b, DNA sample trials), DNA extracted by the coordinator laboratory using a commercial kit was sent to the other laboratories. In RT2a, one male or female individual of each of the four Obsoletus group species collected initially for RT1 was homogenised in 200 μL of 1x PBS and DNA extracted using the commercial DNeasy Tissue and Blood kit (Qiagen, Valencia, CA, USA). From these four extracted DNA samples, one DNA subsample of 11 μL of each species was sent to each participating laboratory with randomised labelling, the day after DNA extraction. During RT2b, each DNA sample was divided into 13 DNA subsamples of 15 μl and one complete set of extractions was sent with randomised labelling to participating laboratories. For the third ring trial (RT3, whole midge trial), each participating laboratory received a panel of 20 whole specimens (16 from the subgenus *Avaritia* and 4 individuals of additional species) which was then processed by each laboratory as they would do normally for routine samples (Table 3). Wings were removed to reduce bias through inadvertent morphological identification. DNA extraction was then conducted on the whole midge without wings. Table 2 details the number and origin of specimens included in each of the ring trials.

**Table 3:** DNA extraction and molecular marker identification methods used by laboratories in each of the ring trials

**Code**	**RT1**		**RT2a**	**RT2b**	**RT3**	
	**Extraction**^**1**^	**Marker used**^**2**^	**Identification**	**Marker used**	**Extraction**^**3**^	**Marker used**
**LabA**	Qiagen Kit	ITS1	ITS1	ND	ND	
**LabB**	Crude lysate	ITS1	ITS1	ND	ND	
**LabC**	Qiagen Kit	ITS1	ITS1	ITS1	Qiagen Kit	COI and ITS1
**LabD**	MN Kit	ITS1	ITS1	ITS1	MN Kit	ITS1
**LabE**	Crude lysate	COI	COI	COI	Qiagen Kit	COI
**LabF**	Roche Kit	COI	COI	ITS1	Roche Kit	ITS1
**LabG**	Chelex	COI	COI	COI	Chelex	COI
**LabH**	ND		ND	ITS1	Squish Kit	ITS1
**LabI**	ND		ND	ITS1	Qiagen Kit	ITS1
**LabJ**	Crude lysate	ITS1 (MA)	ITS1 (MA)	ITS1 (MA)	Chelex	ITS1 (MA)
**LabK**	Crude lysate	ITS2/ITS1	ITS2/ITS1	ITS2/ITS1	IQCasework	ITS2/ITS1
**LabL**	AJ Kit	COI	COI	COI	Qiagen Kit	COI

^1^Qiagen Kit: DNeasy Tissue and Blood kit, Qiagen, USA; MN Kit: NucleoSpin® Tissue, Macherey-Nagel; Roche Kit: High Pure PCR Template Preparation Kit, Roche; Chelex: Chelex 100 chelating ion exchange resin, Bio-Rad; AJ Kit: innuPREP DNA Mini Kit, Analytik Jena; NA: not applicable; ND: not done.

^2^MA: microarray.

^3^Squish Kit: Squish buffer; IQCasework: DNA IQ Casework Sample Kit, Promega.

### Statistical analyses

Separate analyses were performed for each marker as the experimental design did not allow separating the marker effect from the laboratory effect (as each laboratory used only their most commonly implemented technique). Three indicators were defined to assess the accuracy of assays: 1) the sensitivity, as the probability of a correct identification of an Obsoletus group species sample; 2) the lure specificity, as 1 - *p*_*L*_, with *p*_*L*_ the probability of misidentification for a specimen not belonging to the Obsoletus group, as a specimen of the Obsoletus group and 3) the Obsoletus specificity, as 1 - *p*_*O*_, with *p*_*O*_ the probability of misidentification for a specimen from the Obsoletus group, as being identified as another species of the Obsoletus group. Each probability was fitted with a logistic regression model using a quasi-likelihood method accounting for possible over-dispersion in binomial data [41,42]. Fixed effects were the laboratory, the species, the sample (homogenised *Culicoides*; extracted DNA, whole *Culicoides*) and the extraction method. Wald tests were used to assess the effects. All data analyses were performed using the R statistical package [43].

## Results

Twelve laboratories in seven European countries and one northern African country (Belgium, France, Germany, Italy, United Kingdom, Spain, and Tunisia) were involved in these ring trials. Different DNA extraction methods were used as illustrated in Table 3, most of them being commercial kits. Most of laboratories used assays based on ITS1 or COI polymorphisms [37,39]. Only one laboratory used a two-step identification method: first, ITS2-based assay to identify *C. obsoletus*, *C. dewulfi* and *C. chiopterus/C. scoticus* and then, ITS1-based assay to separate *C. chiopterus* and *C. scoticus* (combination of [37,44]). One laboratory used a DNA microarray method based on ITS1 [36]. Two laboratories decided to not take part in RT2 and RT3. Across the 12 participating laboratories a total of 800 molecular assays were performed: 656 on individuals from the subgenus *Avaritia* and 144 on non-Obsoletus group individuals. Identification results were given to all the participating laboratories after each round.

### Morphological identification

Morphological identification differed only for one single specimen out of the 48 cross-identified by the 2 experts for RT1. It was a *C. scoticus* female specimen as confirmed by two different molecular assays identified as *C. scoticus* by one expert and as *C. obsoletus* by the other. All the specimens were included in the ring trials.

### Factors impacting the sensitivity

The overall detection sensitivity for assays using COI and ITS1 was respectively 59.6% and 76.4% (Table 4). The sensitivity was significantly lower for *C. scoticus* (41.7%, *p* < 0.01) than for the other *Avaritia* species which ranged from 61.7% to 70.0% when COI was the target. This difference was not marked when the ITS1-based assay was used. The results from the laboratory using a DNA microarray method based on ITS1 were 100% for all individuals tested (Table 4), whereas those from the laboratory using a two-step assay were 90.6% correct. Regardless of the molecular marker used, the sample type sent to the laboratories impacted upon the sensitivity. Sensitivity was significantly lower when homogenised lysates of ground specimens were sent as template (33.0%, *p* < 0.001), compared to whole *Culicoides* or commercially extracted DNA samples (79.7% and 85.9%, respectively). This was comparable for both molecular markers (COI and ITS1) (Table 4). For ITS1, the sensitivity was lower when crude lysates were used (64.3%, *p* = 0.04) rather than whole insects (81.2%) or DNA samples (86.5%)

**Table 4:** Sensitivity, lure specificity and Obsoletus specificity for assays using COI and ITS1 markers

**Marker**	**Tested effect**		**Lab**	**n**	**Sensitivity**	**n**	**Lure specificity**	**n**	**Obsoletus specificity**
COI	Overall		5	240	59.6	52	92.3	441	99.1
	Laboratory		5	240	65.6 [48.4-81.2]	52	92.3 [85.7-100.0]	441	99.1 [97.6-100.0]
	Species	*C. obsoletus*	5	60	**61.7**	-	-	107	99.1
		*C. scoticus*	5	60	**41.7**	-	-	121	99.2
		*C. dewulfi*	5	60	**70.0**	-	-	103	100.0
		*C. chiopterus*	5	60	**65.0**	-	-	108	98.1
	Sample type	Ground midge	4	112	**33.0**	24	95.8	114	99.1
		Whole midge	4	64	**79.7**	16	100.0	156	99.4
		DNA sample	4	64	**85.9**	12	75.0	171	98.8
	DNA extraction method	Crude lysate	1	28	**53.6**	6	100.0	45	100.0
		Chelex method	1	44	**34.1**	10	100.0	48	97.9
		Commercial kits	3	168	**67.9 [3.6-83.0]**	36	87.5 [83.3-100.0]	345	100.0 [99.0-100.0]
ITS1	Overall		7	288	76.4	64	81.2	795	94.7
	Laboratory		7	288	79.7 [40.6-100.0]	64	100.0 [42.9-100.0]	795	95.0 [83.3-100.0]
	Species	*C. obsoletus*	7	72	81.9	-	-	198	**96.5**
		*C. scoticus*	7	72	80.6	-	-	203	**86.2**
		*C. dewulfi*	7	72	79.2	-	-	197	**97.5**
		*C. chiopterus*	7	72	63.9	-	-	197	**98.9**
	Sample type	Ground midge	4	112	**64.3**	24	70.8	309	90.6
		Whole midge	5	80	**81.2**	20	75.0	198	100.0
		DNA sample	7	96	**86.5**	20	100.0	288	95.5
	DNA extraction method	Crude lysate	1	28	39.3	6	100.0	78	83.3
		Squish buffer	1	16	56.2	4	100.0	27	100.0
		Commercial kits	5	244	81.5 [77.3-100.0]	54	90.0 [20.0-100.0]	661	95.6 [95.0-100.0]
ITS2/ITS1	Overall		1	64	90.6	14	100.0	177	99.4
	Species	*C. obsoletus*	/	33	97.0			90	100.0
		*C. scoticus*	/	32	87.5			94	98.9
		*C. dewulfi*	/	31	100.0			92	100.0
		*C. chiopterus*	/	32	96.9			92	100.0
	Sample type	Ground midge	/	56	92.9	6	100.0	156	100.0
		Whole midge	/	32	93.8	4	100.0	93	98.9
		DNA sample	/	40	100.0	4	100.0	120	100.0
Microarray	Overall		1	64	100.0	14	100.0	192	100.0

Probabilities (expressed as percentages) are written in bold font when the corresponding effect was significantly different from 0 (α = 0.05). Minimum and maximum values are shown in brackets. DNA extraction method is not tested for both ITS2/ITS1 and microarray because one method was used. For the microarray, the number of samples tested is the same as for the ITS2/ITS1.

The DNA extraction method used by the participating laboratories significantly influenced the level of sensitivity (for COI-based assay, *p* < 0.01). Using a crude lysate as the DNA template (meaning no strict DNA extraction method) for one-step assays gave poor sensitivity (53.6% and 39.3%, respectively for COI and ITS1), although this methodology was only utilised by two laboratories (Table 3). The Chelex DNA extraction method gave contrasting results when used on ground midge template (7.1%) compared to whole *Culicoides* (81.3%) (data not shown in Table 4). DNA extraction methods using commercial kits gave moderate to high sensitivity (67.9% and 81.5%, respectively for COI and ITS1), with one exception from one laboratory resulting in low sensitivity (3.6%).

### Factors impacting the lure specificity

No false positive (meaning non-Obsoletus individuals identified as Obsoletus individuals) were detected with the ITS2/ITS1 assay or the microarray. Although not statistically significant, the COI-based assay was more specific than the ITS1-based assay in not identifying lure specimens. The lure specificity was particularly low for the DNA sample/the COI-based assay (75%), and ground and whole midge with the ITS1-based assay (respectively 70.8% and 75%). The COI marker-based assays performed well in not amplifying either non-Obsoletus group *Culicoides* or non-*Culicoides* Ceratopogonidae (92.7% *vs* 90.9%) (data not shown).

### Factors impacting the obsoletus group specificity

Correct identification of Obsoletus group specimens to species was generally achieved (99.1% and 94.7% correctly identified with COI and ITS1 respectively, Table 4). For the ITS1-based assay, the specificity was significantly lower for *C. scoticus* (86.2%, *p* < 0.001) than for the other species (ranging from 96.5% to 98.9%). The Obsoletus specificity was not drastically impacted by the sample type or DNA extraction method for COI-based assay. However, for the ITS1-based assay the specificity was low when used with crude lysate as DNA template for the PCR (83.3%). The Obsoletus specificity was high (100%; ranging from 99 to 100%) when commercial kits for DNA extraction are used with the COI-based assay (Table 4). The overall specificity for the laboratory using the combination of ITS2 and ITS1 was 99.4% and 100% for the ITS1 microarray (Table 4).

One laboratory tested two assays during RT3 (Table 3) on 16 individuals. The sensitivity and Obsoletus specificity was similar (data not shown in Table 4): 81.2% *vs* 87.5% for the COI-based assay and ITS1-based assays respectively; Obsoletus specificity 97.6% *vs* 100% for the COI-based assay and ITS1-based assay respectively.

## Discussion

The use of molecular identification assays for medically important arthropod diagnosis has become routine with the development of PCR-based methodologies, following the increasing dearth of fundamental expertise in classical taxonomy and the difficulty of morphologically identifying each species within a group comprising multiple, closely related, sibling species. Although these assays are widely used in laboratories across the world, to our knowledge, this is the first IMRT conducted in this field, although discrepancies between molecular assays have been demonstrated recently for the *Anopheles gambiae* complex [7]. While comparison across laboratories in the present study is challenging due to subtle methodological differences across the twelve participating laboratories, several broad conclusions can be drawn from the performance trial conducted.

The study initially confirmed that international experts with substantial experience of *Culicoides* morphology (in both cases exceeding 30 years) were able to morphologically differentiate *C. chiopterus* and *C. dewulfi* from *C. obsoletus*/*C. scoticus*, and also to identify specimens within the Obsoletus complex, as either *C. obsoletus* or *C. scoticus* including females with a high degree of accuracy (>95%). As expected, the PCR-based assays used to perform the same task functioned most consistently on samples involving extraction using commercially produced kits. This was most evident in RT1 where both the use of PCR assays directly on crude lysates and following processing with Chelex produced poor results. While the latter result can be explained by the fact that the use of crude lysates as a starting point for the process of extraction was not part of the standardized methodology for Chelex extraction, the use of crude lysates for direct processing is not recommended for *Culicoides* as applied in the current study. The use of Chelex resin is considered to be an effective and cheap option for DNA extraction if used on whole *Culicoides*. The sensitivity is overall moderate to high but is largely influenced by the type of sample (ground midge) and type of DNA extraction (Chelex).

The lure specificity was surprisingly high during RT1 and RT3 using an ITS1-based assay, and RT2 using a COI-based assay. There is an obvious effect of the sample size since a limited number of samples were tested, especially for COI (12 individuals). Moreover, the detailed identification results exposed two laboratories to have false positive scores.

All the in-house assays performed adequately in identifying members of the Obsoletus group, with COI-based identification being marginally more consistent than using ITS1 alone. The COI-based assay was originally developed using samples collected in several European countries (UK, Bulgaria, Italy, Morocco and Greece) [39] whereas the ITS1 assay was based on samples from a single country (France) [37]. Both studies originally investigated the specificity of the primers designed by testing cross-amplification with 14 and 30 *Culicoides* species, respectively. A key additional difference, however, lies in the use of species-specific primers as the ITS1 assay lacks a specific primer for *C. scoticus* in the assay [37], relying on the absence of amplification to identify this species. This was implemented due to a lack of diversity within the marker region that precluded the siting of four differentiating primers and may increase the probability of having both false positive results and misidentification.

The two-stage assays examined during the trial, namely the microarray or the ITS2/ITS1 amplification, gave higher overall specificity compared to one-step assays, with the microarray assay identifying correctly all samples in the three ring trials. It is clear that PCR amplification followed by hybridization (microarray method) [36] or a funnel approach with different markers will increase the specificity. This is clearly at the expense of both time and cost, although these factors are challenging to assess due to differences in the accessibility, price of reagents and labor costs between countries. In addition, these two steps assays were used by only one laboratory in each case and hence variation between users could not be assessed.

Following completion of the current study, several additional assays have been devised to differentiate members of the Obsoletus group [38,40,45-48]. Despite this, the processing of large numbers of *Culicoides* to species level remained extremely rare and is limited to relatively small scale studies [13,49-52]. In this regard, the recent standardization of a real-time PCR assay for high-throughput processing of pools of *C. obsoletus* and *C. scoticus* allowing estimation of proportions of each species has substantial advantages over other available assays [48]. This assay has the potential to be integrated into robotized extraction methods giving a vast potential for rapid processing using methodologies that are familiar to reference laboratory workers. In addition, it is highly cost-effective in allowing the processing of one hundred individuals in a single extraction. A key challenge, however, remains in assessing the degree of repeatability of the assay when used both on a larger number of individuals and in different geographic regions.

A major uncertainty in the identification of *Culicoides* by multiplex assays is the potential presence of cryptic undescribed species within the European fauna which may cross-react with detection systems for known species. Such species have already been highlighted in faunistic inventories although their impact on routine identification has, in general, not been investigated [53]. While many studies have utilized molecular marker sequencing in particular to assess the *Culicoides* fauna across Europe [37,39,54], coherent estimations of likely species diversity and paired DNA sequence/morphological voucher specimen collections remain in their infancy.

## Conclusion

To conclude, this study has illustrated that molecular identification assays are accurate tools for species diagnosis, though precautions are required during several steps from the storage of the specimens/samples to the PCR amplification step to ensure correct identification. Based on the results presented in this study, some recommendations are suggested: (i) when samples need to be sent for molecular analysis, one should prefer to send them as whole midge stored in 70% alcohol or as DNA samples extracted with commercial kits; (ii) although expensive when a large number of samples need to be identified, commercial DNA extraction kits allow high sensitivity and specificity, (iii) for molecular identification of the four targeted species, the COI-based assay showed higher specificity of the one-step molecular identification assays, and (iv) the development of molecular identification assays is important and must always include sensitivity and specificity assessment. Primer design should be conducted on sequence alignments that include several conspecific specimens from different sites over a wide geographic range. Wide-scale IMRTs are also recommended in assessing the reproducibility and robustness of molecular assays using different groups of researchers and a variety of different in-house procedures. Finally, although we emphasize the importance of molecular identification assays, we stress that there remains an urgent need to sustain traditional taxonomy based on morphology. Morphological identification expertise for arthropods of medical importance needs to be maintained to strengthen the systematics and taxonomy of these groups while new molecular tools are required to process large scale surveillance specimens across countries.

## Competing interests

The authors declare that they have no competing interests.

## Authors’ contributions

CG, TB, ThB, CCS designed the study. JCD, RM, IR, AP, LG prepared the sample sets. SC, IR, AP, LG, NG, JB, MM, DBB, MG, FM, NP, SS, SH, JHC, JL, DG, GDD, VSM, JS, HK, BH, KL, DW participated to the assays. TB and RL analyzed the data. CG, TB, RM, SC, ThB, CCS wrote the manuscript, which was revised and approved by all the co-authors.
